# 7^th^ Brazilian Guideline of Arterial Hypertension: Chapter 9 - Arterial Hypertension in pregnancy

**DOI:** 10.5935/abc.20160159

**Published:** 2016-09

**Authors:** MVB Malachias, CEP Figueiredo, N Sass, IC Antonello, MR Torloni, MRFL Bortolotto

## Epidemiology

The hypertensive syndromes of pregnancy cause expressive maternal and fetal morbidity and mortality. There is no accurate information on the incidence of preeclampsia (PE), but it is estimated to affect 4% of gestations. In Brazil, the incidence of PE is 1.5 %, while the incidence of eclampsia is 0.6%.^1^ More developed areas have an incidence of eclampsia of 0.2%, with a maternal death rate of 0.8%, while for less favored regions those indices are 8.1% and 22%, respectively.^2^ A population-based study shows AH in 7.5% of the gestations in Brazil, with 2.3% of PE and 0.5% of superimposed PE.^3^ Arterial hypertension during pregnancy accounts for 20% to 25% of all causes of maternal death, and data from SUS show a trend towards stagnation.^4^

## Classification

This guideline recommends the American College of Obstetricians and Gynecologists's (ACOG) classification of hypertension in pregnancy^5^ (Chart 1). (GR: IIb; LE: C).

**Chart 1:** Classification of hypertension in pregnancy.

Preeclampsia – Eclampsia
Chronic AH (any etiology)
Chronic AH with overlapping PE
Gestational hypertension

## Concept and diagnosis criteria

Arterial hypertension in pregnancy is defined as the presence of SBP ≥ 140 mm Hg and/or DBP ≥ 90 mm Hg, considering the fifth Korotkoff sound, confirmed by another measurement after 4 hours. For BP measurement, the patient should be ideally sitting, or, alternatively, in the lateral decubitus position. Proteinuria is considered: a) protein ≥ 300 mg in 24-hour urine; b) urine albumin/creatinine ratio (UACR) ≥ 0.3 mg/mg in an isolated sample; c) positive reagent strip test in at least two samples (quantification is suggested).

Preeclampsia is defined as the presence of AH after the 20th gestational week, associated with significant proteinuria. In the absence of significant proteinuria, the diagnosis can be based on the presence of: headache, blurred vision, abdominal pain, low blood platelet count (< 100,000/mm³), elevation of liver enzymes (twice the baseline level), kidney impairment (creatinine > 1.1 mg/dL or twice the baseline level), pulmonary edema, visual or cerebral disorders, scotomas, and seizure. Eclampsia is defined as the presence of grand mal seizure in a pregnant woman with PE.

Chronic AH is defined by the detection of AH prior to pregnancy or before the 20th week of gestation. Preeclampsia might overlap. Gestational hypertension is characterized by AH after the 20th week of gestation without proteinuria.

Some clinical conditions increase the risk of PE dramatically. Severe PE should be considered in the presence of: SBP ≥ 160 or DBP ≥ 110 mm Hg; low blood platelet count; TGP twice the baseline level; persistent epigastric or right hypochondrial pain; acute kidney injury (AKI - creatinine > 1.1 mg/dL or twice the baseline level); pulmonary edema; visual or cerebral symptoms.^5^

## Preeclampsia prevention

Regarding PE prevention, there is no unequivocally effective strategy for all pregnant women. Calcium supplementation (> 1 g/day) is not recommended for pregnant women with normal intake of that ion^6^ (GR: III; LE: A), but to those with low calcium intake and at intermediate and increased risk for PE.^6^ (GR: I; LE: A). Low doses of acetylsalicylic acid (75-150 mg/day) at the end of the first gestational trimester can be useful for primary prevention of PE in pregnant women at intermediate and increased risk for PE.^7,8^ (GR: IIa; LE: B). That use, however, is not recommended in the absence of risk.^8^ (GR: III; LE: A). Calcium supplementation (> 1 g/day) is associated with a reduction in the risk for PE, prematurity and a lower risk of gestational-hypertension-related death, particularly in women with a low-calcium diet (< 600 g).^6^ For women at risk for PE, clinical trials have suggested a significant protective effect of the daily acetylsalicylic acid use.^7^ Low acetylsalicylic acid doses reduce the risk of PE by 17%, with a decrease in the fetal death risk of 14% and in the prematurity risk of 8%. Daily doses of 75-150 mg seem safe.^8^ Acetylsalicylic acid at low doses should be considered for primary prevention of women at high risk and should be initiated at the end of the first trimester.^9^

## Nonpharmacological treatment

For persistent AH > 150 mm Hg for more than 15 minutes, NPT alone should not be used to prevent irreversible neurological damage.^10^ (GR: III; LE: B). Systolic BP > 155 mm Hg, especially > 160 mm Hg, is detected immediately before stroke.^11^ Severe diastolic AH (DAH: > 105 or 110 mm Hg) does not develop before most strokes of pregnant women with severe PE.^11^ To avoid maternal deaths, SBP > 150-160 mm Hg should indicate urgent treatment.^12^

Relative rest at hospital or day-hospital with monitoring is suggested for PE. (GR: IIa; LE: B). Hospitalization should be indicated to pregnant patients with severe AH. (GR: I; LE: B). A systematic review has shown no difference in outcomes between strict rest and relative rest for pregnant women with AH and proteinuria. Relative rest at hospital, as compared with routine house activity, reduces the risk for severe AH; however, data do not support a clear recommendation. Rest is not routinely indicated for gestational hypertension.^13^ Prenatal care units and hospitals have similar clinical outcomes for mothers and newborns, but women might prefer day-hospitals.^14^

Although there is no indication for specific care during hospitalization, maternal and fetal monitoring is required. Blood pressure should be periodically measured, with daily weight and diuresis assessment, and patients should be instructed about premonitory signs. Laboratory tests, such as hemogram with platelet count, liver enzymes, uric acid, creatinine and proteinuria, should be performed once to twice a week. Fetal follow-up comprises assessment of growth, movements, well-being and biophysical profile, as well as US.

## Expectant management

Expectant management is not recommended after the 36th gestational week for women with gestational hypertension.^15^ (GR: III; LE: B). Expectant management is suggested between the 34th and 36th gestational weeks for stable women, without clinical worsening or severe hypertension.^16^ (GR: IIa; LE: B). Premature delivery for patients with PE can be associated with decreased mortality. The ideal delivery time, before the 32nd-34th weeks, is a dilemma because of the uncertainty in the balance between maternal safety (end of pregnancy) and fetal maturity (expectant).^17^ After the 34th week, survival is high and the baby and placenta delivery is effective in developed countries.^17^

The HYPITAT study has compared delivery induction versus expectant monitoring for severe AH or mild PE after the 36th week.^15^ Women in the intervention group had a 29% lower risk of worse maternal outcome, without affecting neonatal outcome, suggesting that expectant treatment after 36 weeks is not indicated.^15^ In the HYPTAT-II study, with non-severe AH between the 34^th^ and 37^th^ weeks, expectant management increased maternal risk as compared to immediate delivery, but decreased the occurrence of neonatal respiratory distress syndrome. In that situation, immediate delivery is not justified, and expectant monitoring until the clinical situation worsens should be considered.^16^

## Pharmacological treatment

Urgent pharmacological treatment is indicated in severe AH (SBP > 155-160 mm Hg)^10,11^ and presence of premonitory signs. (GR: I; LE: B). The treatment of severe AH in emergency situations can be performed with intravenous (IV) hydralazine (5 mg, repeat 5-10 mg IV every 30 minutes until the maximum of 20 mg). In exceptional situations, such as acute pulmonary edema (APE) and refractory severe SAH, the use of sodium nitroprusside (SNP) should be the preferential option for urgent BP control.^18^ Oral administration of rapid-acting nifedipine (5 mg every 30 minutes) is an alternative, but associated complications have been reported.^19^ Although sublingual nifedipine is not indicated in other forms of hypertensive crisis (HC), it is an alternative in gestational hypertension. Its use for hypertensive emergency (HE), however, has been considered bad practice, harmful to the patient in a report by the São Paulo Regional Medical Board.

Pharmacological treatment should be initiated whenever BP is > 150/100 mm Hg,^12^ aiming at maintaining it 130-150/80-100 mm Hg. (GR: IIa; LE: B). For stable patients with PE, not requiring immediate delivery, oral antihypertensive treatment is indicated. Treatment with antihypertensive agents reduces the risk for severe AH, but not the risk for PE, restricted intrauterine growth, placental abruption and neonatal outcomes.^20^ Treatment to meet the DBP target of 85 mm Hg as compared to 100 mm Hg showed neither maternal nor obstetric benefit, except for the less frequent occurrence of severe AH in the group with stricter control.^21^

The choice of the antihypertensive drug depends on the attending physician's experience and familiarity with the drug chosen and its possible side effects.^22^ (GR: IIb; LE: B). The use of ACEIs, ARBs and direct renin inhibitors is contraindicated in pregnancy (GR: I; LE: B), and atenolol and prazosin should be avoided.^22,23^ (GR: IIa; LE: B). In Brazil, the available oral drugs usually used are methyldopa, BBs (except atenolol), hydralazine and CCBs (nifedipine, amlodipine and verapamil). Atenolol is associated with a reduction in fetal growth, and prazosin can cause natimortality.^24-26^ For PE, the prescription of a DIU is usually avoided; thiazide DIUs, however, can be continued in pregnant women with chronic AH (CAH),^27^ as long as they do not cause volume depletion.

Magnesium sulfate is recommended for eclampsia prevention and treatment. (GR: I; LE: B). In case of HE or of hypertensive urgency (HU) requiring hospitalization, intensive monitoring, preterm delivery and parenteral administration of antihypertensive drugs, the IV administration of magnesium sulfate, considered the drug of choice to prevent and treat eclampsia, is recommended.^5,28-30^ Magnesium sulfate is administered at an attack dose of 4-6 g IV for 10-20 minutes, followed by infusion of 1-3 g/h, usually for 24 hours. If the seizure reoccurs, 2-4 g can be administered IV. Deep intramuscular administration of 10 g (5 g in each gluteus), followed by intramuscular 5 g every 4 hours for 24 hours is an alternative. Magnesium sulfate is indicated during labor for patients with severe PE. Magnesium sulfate administration should continue for up to 24 hours after seizure, imminent eclampsia signs and delivery. Its administration should be generous to patients with PE, preferably before the administration of rapid-acting antihypertensive drugs to patients, in whom the possibility of eclampsia cannot be ruled out.

## Other important aspects

Severe AH, per se, is not an indication for C section. In the presence of stable maternal clinical findings, good fetal vitality and lack of other C section indications, pregnancy termination can occur by delivery induction, always considering maternal clinical condition and fetal vitality during the procedure.^5,29^ Labor analgesia is recommended with local-regional techniques (peridural or combined analgesia). Severe thrombocytopenia contraindicates anesthesia with lumbar puncture, and, if C section is required, it should be performed under general anesthesia. Invasive central monitoring is reserved to cases with hemodynamic instability (respiratory failure, disseminated intravascular coagulation related to placental abruption, or HELLP syndrome).^5^

## Antihypertensive treatment in lactating women

Chart 2 shows the antihypertensive drugs available in Brazil considered safe, moderately safe and not recommended.^31,32^ (GR: IIb, LE: C).

**Chart 2:** Safety of the infant breastfed by a lactating woman on antihypertensive drugs.

Drugs	Recommendation
DIUs: hydrochlorothiazide and spironolactone. Adrenergic inhibitors: alpha-methyldopa and propranolol. Vasodilators: hydralazine and minoxidil. CCBs: verapamil, nifedipine, nimodipine and nitrendipine. ACEIs: benazepril, captopril and enalapril.	Safe
DIUs: indapamide, furosemide and triamterene. Adrenergic inhibitors: atenolol, bisoprolol, carvedilol, metoprolol, sotalol. CCBs: amlodipine, isradipine, nisoldipine. ACEIs: lisinopril, ramipril. ARBs: candesartan and olmesartan. Telmisartan after the perinatal period.	Moderately safe
Adrenergic inhibitors: reserpine, prazosin and terazosin. ARBs: telmisartan, in the perinatal period; valsartan.	Potentially harmful

DIUs: diuretics; CCBs: calcium-channel blockers; ACEIs: angiotensin-converting-enzyme inhibitors; ARBs: angiotensin-receptor blockers.
